# Comparative Evaluation of Urinary PCA3 and TMPRSS2: ERG Scores and Serum PHI in Predicting Prostate Cancer Aggressiveness

**DOI:** 10.3390/ijms150813299

**Published:** 2014-07-30

**Authors:** Lucile Tallon, Devillier Luangphakdy, Alain Ruffion, Marc Colombel, Marian Devonec, Denis Champetier, Philippe Paparel, Myriam Decaussin-Petrucci, Paul Perrin, Virginie Vlaeminck-Guillem

**Affiliations:** 1 Medical Unit of Molecular Oncology and Transfer, Department of Biochemistry and Molecular Biology, University Hospital of Lyon Sud, Hospices Civils of Lyon, Chemin du Grand Revoyet, 69495 Pierre Bénite, France; E-Mails: lu.tallon@gmail.com (L.T.); ldevillier@gmail.com (D.L.); 2 Department of Medicine and Pharmacy, Faculty of Lyon 1 University, 8 Avenue Rockefeller, 69373 Lyon, France; E-Mails: alain.ruffion@chu-lyon.fr (A.R.); marc.colombel@chu-lyon.fr (M.C.); marian.devonec@chu-lyon.fr (M.D.); philippe.paparel@chu-lyon.fr (P.P.); myriam.decaussin-petrucci@chu-lyon.fr (M.D.-P.); paul.perrin@chu-lyon.fr (P.P.); 3 Department of Urology, University Hospital of Lyon Sud, Hospices Civils of Lyon, Chemin du Grand Revoyet, 69495 Pierre Bénite, France; E-Mail: denis.champetier@chu-lyon.fr; 4 Institute of Functional Genomics of Lyon, CNRS UMR5242, Lyon 1 University-CNRS-INRA, 46 Allée d’Italie, 69364 Lyon, France; 5 Department of Urology, E. Herriot Hospital, Hospices Civils of Lyon, Place d’Arsonval, 69003 Lyon, France; 6 Joint Research Unit INSERM 1033, Lyon 1 University, Avenue Rockefeller, 69373 Lyon, France; 7 Department of Pathology, University Hospital of Lyon Sud, Hospices Civils of Lyon, Chemin du Grand Revoyet, 69495 Pierre Bénite, France; 8 Centre de Recherche en Cancérologie de Lyon, U1052 INSERM, UMR CNRS 5286, Université Lyon I, Centre Léon Bérard, Rue Laennec, 69008 Lyon, France

**Keywords:** urine biomarker, prostate cancer, PCA3, TMPRSS2-ERG gene fusion, PHI, radical prostatectomy, aggressiveness

## Abstract

It has been suggested that urinary PCA3 and TMPRSS2:ERG fusion tests and serum PHI correlate to cancer aggressiveness-related pathological criteria at prostatectomy. To evaluate and compare their ability in predicting prostate cancer aggressiveness, PHI and urinary PCA3 and TMPRSS2:ERG (T2) scores were assessed in 154 patients who underwent radical prostatectomy for biopsy-proven prostate cancer. Univariate and multivariate analyses using logistic regression and decision curve analyses were performed. All three markers were predictors of a tumor volume ≥0.5 mL. Only PHI predicted Gleason score ≥7. T2 score and PHI were both independent predictors of extracapsular extension (≥pT3), while multifocality was only predicted by PCA3 score. Moreover, when compared to a base model (age, digital rectal examination, serum PSA, and Gleason sum at biopsy), the addition of both PCA3 score and PHI to the base model induced a significant increase (+12%) when predicting tumor volume >0.5 mL. PHI and urinary PCA3 and T2 scores can be considered as complementary predictors of cancer aggressiveness at prostatectomy.

## 1. Introduction

Although Prostate-Specific Antigen (PSA) assay is not recommended for prostate cancer (PCa) screening (review in [1]), it is largely used for early detection and contributes to increase the incidence of PCa. It adversely, also, led to increase in the diagnosis of clinically insignificant tumors (overdiagnosis) and their early treatment (overtreatment) [1]. Efforts are therefore currently made to develop biomarkers able to identify cancers that require intervention and to help physicians in the choice of focal treatments. Prostate cancer gene 3 (PCA3), TMPRSS2:ERG gene fusion and PHI are among the most promising biomarkers that could complement PSA for early PCa diagnosis.

PCA3 gene products a non-coding RNA, whose function is largely unknown. Although no or weak expression was reported in non-malignant prostate tissues, PCA3 overexpression was observed in up to 95% of PCas [2]. A urinary test has been proposed for a decade [3] and is now commercially available as a kit that specifically captures (magnetic beads), amplifies (transcription-mediated amplification), detects (hybridization protective assay), and quantifies PCA3 RNA copies in urine samples obtained after an attentive digital rectal examination (DRE) from patients with PCa suspicion [4]. Numerous clinical studies established the diagnostic potential of PCA3 test as a means to help physicians in deciding prostate cancer biopsies with a better specificity than serum PSA (revue *in* [5,6]). The test received in 2012 FDA agreement in patients 50 years of age or older who have had one or more previous negative biopsies and for whom a repeat biopsy would be recommended.

Fusion of the androgen-regulated TMPRSS2 gene promoter (transmembrane protease, serine 2) to the ETS transcription factors ERG or ETV1 was described as recurrent in the majority of PCas [7]. Subsequent studies confirmed ETS gene fusions in about 50% of PSA screened PCas [8]. TMPRSS2:ERG fusions represent about 90% of all ETS gene fusions and are typically detected at the chromosomal level by fluorescence *in situ* hybridization [8]. The subsequent overexpression of the TMPRSS2:ERG transcript (*in situ* hybridization or RT-PCR) is reported as highly specific for the presence of cancerous tissue in prostate tissue-based studies [8,9]. Similar to the PCA3 transcript, TMPRSS2:ERG mRNA is detectable in urine [10,11]. A specific urinary test, the T2 test, recently developed on the basis of the same methodology than the PCA3 test, provides quantification of the amount of TMPRSS2:ERG transcripts [12]. Recent results demonstrated that the combination of PCA3 and T2 tests enhances the utility of PSA for predicting PCa risk [12].

Prostate Health Index (PHI) is based on the assessment of both total serum PSA, its free fraction and a precursor isoform named [-2]proPSA. Some papers recently suggested that it could improve discrimination both between patients with and without PCa and it therefore received FDA agreement in 2012. It could also discriminate between cases of clinically significant or indolent cancer [13,14,15,16,17]. Combination of PHI with PCA3 and/or T2 scores was also acknowledged as useful [18,19,20,21,22].

Whether PHI and PCA3 and T2 tests allow distinction between significant and insignificant PCas is still matter of debate. Since the first report based on prostatectomy specimens [23], correlation between PCA3 score and tumor volume has been confirmed by subsequent studies [24,25,26,27]. By contrast, although also early reported [23], correlation with Gleason score (GS) has not been consistently observed in more recent papers [27,28,29,30,31]. We and others reported correlation between PCA3 score and number of tumor foci [27,32], while others highlighted a link with pT stage [25,26,33] or perineural extension [34]. Correlations of T2 score with GS, pT stage or tumor volume have been inconsistently found [12,30,32,35]. A recent study reported a correlation between PHI and GS, pT stage and tumor volume [36]. Biological predictors of actual GS, tumor volume, pT stage, or number of foci (PCa aggressiveness) could have significant clinical applicability in appropriately selecting patients to the most appropriate treatment: active surveillance, focal treatment or radical treatment. The aim of our study was to evaluate and compare, in a prospective cohort of patients undergoing radical prostatectomies for biopsy-proven PCa, the ability of PHI and PCA3 and T2 scores to predict GS, low tumor volume, multifocality and extracapsular extension at final pathology.

## 2. Results and Discussion

### 2.1. Baseline Characteristics of the Studied Patients

Baseline characteristics of the 154 included patients and pathological findings at biopsies and prostatectomies are shown in Table 1 and Table 2. Median patient age was 64 years. Preoperative median PSA was 6.5 ng/mL. Pathologic GS at prostatectomy was ≥7 in 132 patients (86%), and 48 (31%) were diagnosed with extracapsular extension. Within the 63 patients with biopsy GS ≤6, 43 (68%) showed an upgrading of the GS at final pathology. Tumor volume was ≥0.5 mL in 134 patients (88%).

**Table 1:** Baseline characteristics of the 154 patients who underwent radical prostatectomy.

Baseline Characteristics	Number of Patients (%) or Median (IQR)
Age (years)	64 (58–66)
Gleason score at biopsy	6: *n* = 63 (41%) 7: *n* = 79 (51%) 8: *n* = 12 (8%)
tPSA (ng/mL)	6.5 (5.0–9.9)
fPSA (ng/mL)	0.9 (0.6–1.2)
%fPSA (%)	12.5 (10.5–16.1)
%p2PSA (%)	13.8 (10.2–19.7)
PHI	42.15 (33–56)
PCA3 score	45 (23–87)
T2 score	34 (5–138)

Abbreviations: IQR: interquartile range; PCA3: prostate cancer gene 3; PSA: prostate-specific antigen; tPSA: total serum PSA; fPSA: free serum PSA; %fPSA: fPSA-to-tPSA ratio; %p2PSA: p2PSA-to-tPSA ratio; PHI: Prostate Health Index; SD: standard deviation; T2: TMPRSS2:ERG fusion gene.

**Table 2:** Pathological characteristics of the tumors in prostatectomy specimens and correlations with serum PSA (Prostate-Specific Antigen), PHI (Prostate Health Index), urinary PCA3 (Prostate Cancer gene 3) and T2 (TMPRSS2:ERG) scores.

Pathological Characteristics Prostatectomy *(Number of Patients)*	Number of Patients (%)	Serum PSA		PCA3 Score		T2 Score		PHI	
		Median (IQR)	*p* Value	Median (IQR)	*p* Value	Median (IQR)	*p* Value	Median (IQR)	*p* Value
Pathological T stage *n* = *154*	pT2 *n* = 106 (69%)	6.5 (5.0–9.0)	0.467	47 (23–82)	0.724	27 (5–106)	**0.027**	41 (32–53)	0.086
	≥pT3 *n* = 48 (31%)	6.4 (5.2–10.9)		40 (25–111)		52 (10–352)		47 (34–59)	
Gleason stage *n* = *154*	G6 *n* = 22 (14%)	6.5 (4.6–11.0)	0.739	47 (23–81)	0.897	38 (6–148)	0.123	34 (31–40)	0.013
	G ≥ 7 *n* = 132 (86%)	6.5 (5.2–9.3)		43 (23–90)		35 (7–151)		44 (34–57)	
Total tumor volume (mL) ^a^ *n* = *153*	<0.5 *n* = 19 (12%)	6.2 (4.1–10.1)	0.276	23 (15–47)	0.004	14 (1–105)	0.090	32 (27–42)	0.002
	≥0.5 *n* = 134 (88%)	6.5 (5.1–9.3)		48 (25–94)		37 (6–154)		44 (34–57)	
Number of tumor foci *n* = *154*	Uni *n* = 41 (27%)	6.1 (4.3–11.0)	0.230	27 (19–49)	0.002	55 (8–178)	0.299	41 (33–52)	0.410
	Multi *n* = 113 (73%)	6.6 (5.4–9.2)		51 (27–94)		33 (3–127)		43 (33–57)	
Blood embols *n* = *154*	No *n* = 148 (96%)	6.5 (5.0–9.1)	0.030	44 (23–85)	0.253	35 (5–138)	0.709	42 (33–55)	0.009
	Yes *n* = 6 (4%)	12.9 (8.6–18.1)		103 (25–148)		20 (2–164)		79 (46–140)	
Positive resection margins *n* = *154*	R0 *n* = 121 (79%)	6.3 (5.0–8.8)	0.029	42 (23–82)	0.241	36 (5–137)	0.620	40 (32–53)	0.007
	R1 *n* = 33 (21%)	7.8 (5.9–14.1)		61 (26–110)		29 (3–214)		50 (41–65)	

Abbreviations: IQR: interquartile range; PCA3: prostate cancer gene 3; PHI: Prostate Health Index; PSA: prostate-specific antigen; T2: TMPRSS2:ERG fusion gene; a: median total tumor volume = 2.2 (IQR: 0.9–4.1).

Expectedly, serum PSA correlated with PHI. No correlation was found between PSA or PHI and PCA3 or T2 score. By contrast, PCA3 and T2 scores correlated with each other. Only PCA3 positively correlated with age. Serum PSA was correlated with prostate volume as determined by preoperative TRUS and with prostate weight at prostatectomy. No other correlation was found between the biomarkers and prostate volume or prostate weight at prostatectomy.

### 2.2. Correlations between Biological Biomarkers and Pathological Findings

**Figure 1:**
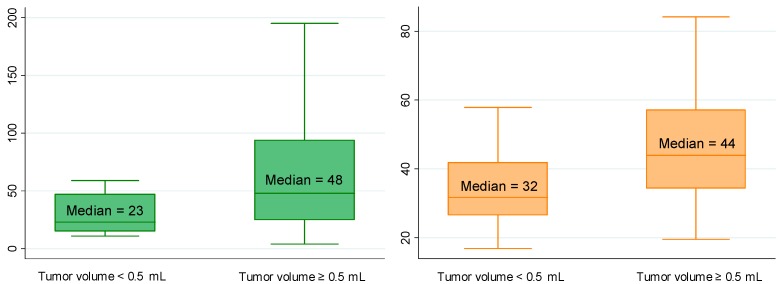
Median PCA3 score (*p* = 0.004) and PHI (*p* = 0.002) according to tumor volume. Biological and pathological findings in 154 men who underwent radical prostatectomies for prostate cancer. Note: The boxes are bordered at the 25th (p25) and the 75th (p75) percentiles (the so-called inter-quartile range IQR) with the median line at the 50th percentile. Whiskers extend from the box to the upper and lower adjacent values. The upper adjacent value is defined as the largest data point ≤(p75 + 1.5 × IQR). The lower adjacent value is defined as the smallest data point ≥(p25 − 1.5 × IQR).

**Figure 2:**
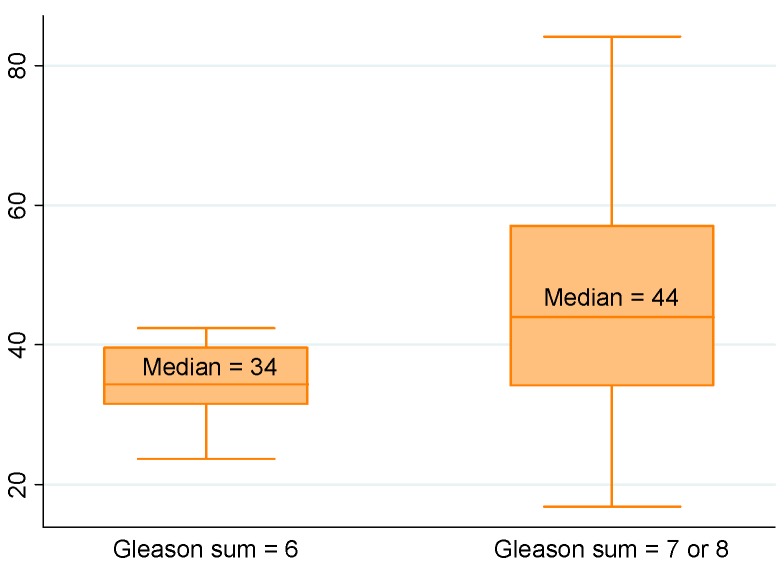
Median PHI according to Gleason sum (*p* = 0.013). Biological and pathological findings in 154 men who underwent radical prostatectomies for prostate cancer. (See the note in the legend of the Figure 1 for complete explanation of how to read the figure).

**Figure 3:**
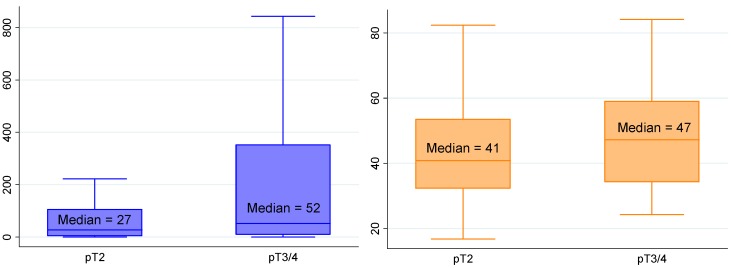
Median T2 score (*p* = 0.027) and PHI (*p* = 0.086) according to extracapsular extension. Biological and pathological findings in 154 men who underwent radical prostatectomies for prostate cancer. (See the note in the legend of the Figure 1 for complete explanation of how to read the figure).

**Figure 4:**
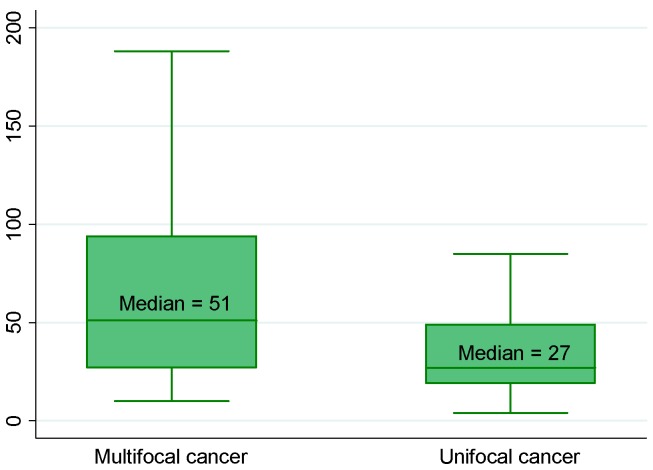
Median PCA3 score according to number of prostate cancer foci (*p* = 0.002). Biological and pathological findings in 154 men who underwent radical prostatectomies for prostate cancer.(See the note in the legend of the Figure 1 for complete explanation of how to read the figure).

Using univariate linear regression, PCA3 score and PHI, as well as T2 score although less markedly, were predictors of a tumor volume ≥0.5 mL (Table 2 and Table 3; Figure 1). Only PHI predicted GS at prostatectomy ≥7 (Figure 2). T2 score and PHI were both predictors of extracapsular extension (≥pT3) (Figure 3), while multifocality was only predicted by PCA3 score (Figure 4). Positive resection margins were predicted by both serum PSA and PHI (Table 3). Multivariate analyses showed that PHI was the only independent predictor of tumor volume ≥0.5 mL (Table 3). T2 score and PHI proved to be both independent predictors of extracapsular extension.

**Table 3:** Multivariate analyses evaluating the ability of clinico-biological variables to predict major outcomes at prostatectomy.

Pathological Characteristics	Serum PSA	Urinary PCA3 Score	Urinary T2 Score	PHI
Tumor volume				
Univariate analysis	*p* = 0.240	*p* = 0.004	*p* = 0.025	*p* = 0.004
Multivariate analysis	-	*p* = 0.076	*p* = 0.249	*p* = 0.020
Gleason sum				
Univariate analysis	*p* = 0.658	*p* = 0.922	*p* = 0.168	*p* = 0.028
Multivariate analysis	-	-	-	-
pT stage				
Univariate analysis	*p* = 0.231	*p* = 0.328	*p* = 0.005	*p* = 0.038
Multivariate analysis	-	-	*p* = 0.026	*p* = 0.042
Multifocality				
Univariate analysis	*p* = 0.704	*p* = 0.014	*p* = 0.413	*p* = 0.814
Multivariate analysis	-	-	-	-
Positive margins				
Univariate analysis	*p* = 0.006	*p* = 0.580	*p* = 0.074	*p* = 0.003
Multivariate analysis	*p* = 0.357	-	-	*p* = 0.131

Abbreviations: PCA3: prostate cancer gene 3; PHI: Prostate Health Index; PSA: prostate-specific antigen; T2: TMPRSS2:ERG fusion gene.

### 2.3. Integration of Biological Biomarkers into Predictive Models

We next assessed whether the biological biomarkers significantly improve predictive accuracy of preoperative predictive models. Base model was defined by including age (continuous variable), DRE findings (suspicious *vs.* non suspicious), serum PSA (continuous variable) and GS at biopsy (6 *vs.* ≥7). We then assessed the additional value of those biomarkers found to be significant in above-cited analyses. The predicted accuracy of the base model in predicting GS ≥7 at prostatectomy was estimated by an AUC of 81%. The addition of PHI (the only significant biomarker) improved the AUC to 86% but this 5% difference did not reach statistical significance (Table 4).

**Table 4:** Prediction of Gleason sum ≥7 at prostatectomy.

Predictors	Univariate Analysis			Multivariate Analysis	
				Base Model	Base Model + PHI
	OR (95% CI)	*p* Value	AUC ((95% CI)	OR ((95% CI)	OR ((95% CI)
Age	1.067 (0.989–1.153)	0.099	62.5% (50.1–74.8)	1.018 (0.935–1.110)	1.047 (0.954–1.150)
DRE findings	1.705 (0.471–6.177)	0.393	53.8% (45.7–61.9)	0.959 (0.215–4.275)	1.413 (0.268–7.438)
Serum total PSA	1.022 (0.926–1.127)	0.658	52.2% (38.0–66.4)	0.966 (0.856–1.091)	0.867 (0.738–1.018)
Biopsy Gleason sum	20.698 (4.626–92.615)	<0.0001	79.2% (71.8–86.5)	21.505 (4.418–104.677)	18.839 (4.018–88.335)
PHI	1.030 (0.998–1.062)	0.028	66.6% (54.4–78.7)	-	1.049 (1.002–1.097)
AUC (multivariate models)				81.3% (71.1–91.5)	86.1% (79.0–93.1)
Gain in predictive accuracy *				-	+4.8
*p* value *				-	NS

All variables were treated as continuous variables except DRE (digital rectal examination; suspicious *vs.* non suspicious) and Gleason sum at biopsy (6 *vs.* ≥7). *: as compared with the base model. AUC: area under receiver operating curves; NS: not significant; OR: odds ratio; PHI: Prostate Health Index; PSA: prostate-specific antigen.

The predicted accuracy of the base model in predicting tumor volume ≥0.5 mL at prostatectomy was estimated by an AUC of 69%. The addition of PCA3 score, T2 score and PHI improved the AUC to 74%, 72%, and 76%, respectively. No difference reached statistical significance (Table 5). The addition of PHI simultaneously to PCA3 score provided a significant 12% increase in AUC. The combination of the three biomarkers to the base model also significantly provided a significant 14% increase in AUC although T2 score appeared poorly informative (Table 5). Increased AUCs were also obtained—although non significantly—when evaluating the additional value of incorporating PHI into the base model to predict positive resection margins, incorporating PHI or T2 score into the base model to predict extracapsular extension, or incorporating PCA3 score to the base model to predict multifocality.

**Figure 5:**
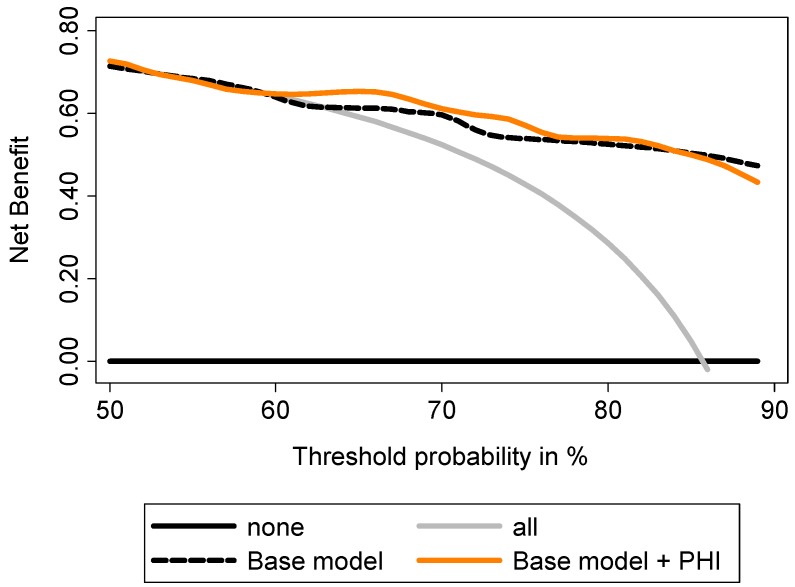
Evaluation of the ability of PHI to predict Gleason sum ≥7 using decision curve analysis. Base model: age, DRE findings, total serum PSA and Gleason sum at biopsy.

**Figure 6:**
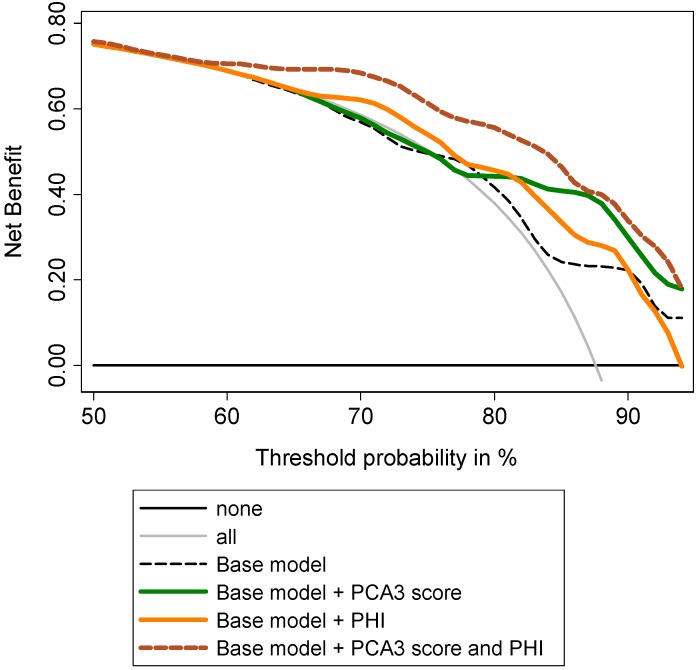
Evaluation of the ability of PHI and PCA3 score to predict tumor volume ≥0.5 mL using decision curve analysis. Base model: age, DRE findings, total serum PSA, and Gleason sum at biopsy.

**Figure 7:**
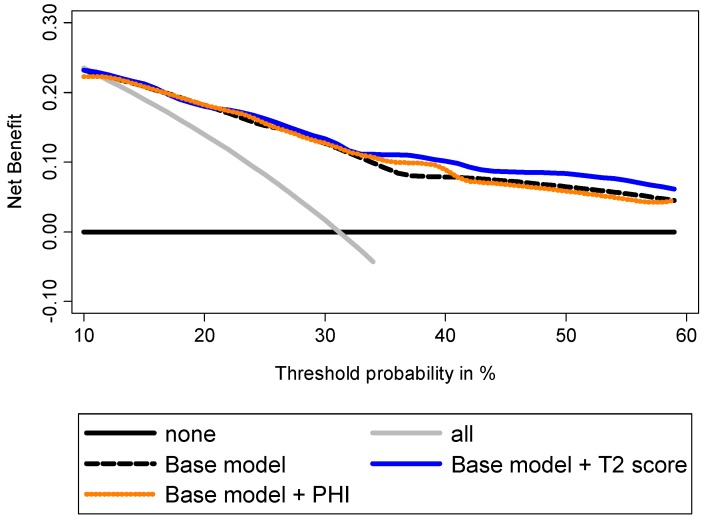
Evaluation of the ability of PHI and T2 score to predict extracapsular extension using decision curve analysis. Base model: age, DRE findings, total serum PSA, and Gleason sum at biopsy.

Decision curve analyses (DCA) showed a higher benefit in incorporating PHI to the base model to predict GS ≥7 at prostatectomy (Figure 5). To predict tumor volume ≥0.5 mL, DCA showed that addition of PHI to the base model provided a higher benefit, especially for intermediate prostate cancer risk (Figure 6). Addition of PCA3 score was beneficial for high prostate cancer risk. Addition of both biomarkers provided the best increase in clinical benefit (Figure 6). To predict extracapsular extension, the addition of T2 score to the base model provided the highest benefit when compared to PHI (Figure 7), while combination of both biomarkers was not beneficial (not shown).

**Table 5:** Prediction of tumor volume ≥0.5 mL at prostatectomy.

Predictors	Univariate Analysis			Multivariate Analysis							
				Base Model	Base Model + PCA3 Score	Base Model + T2 Score	Base Model + PHI	Base Model + PCA3 and T2 Scores	Base Model + PCA3 Score + PHI	Base Model + T2 Score + PHI	Base Model + PCA3 and T2 Scores + PHI
	OR (95% CI)	*p* Value	AUC (95% CI)	OR (95% CI)	OR (95% CI)	OR (95% CI)	OR (95% CI)	OR (95% CI)	OR (95% CI)	OR (95% CI)	OR (95% CI)
Age	1.091 (1.007–1.183)	0.035	67.2% (55.3–79.2)	1.096 (1.008–1.193)	1.066 (0.977–1.164)	1.091 (1.001–1.189)	1.119 (1.021–1.227)	1.070 (0.980–1.169)	1.087 (0.988–1.196)	1.110 (1.011–1.220)	1.089 (0.988–1.198)
DRE findings	1.409 (0.383–5.177)	0.595	52.6% (43.4–61.7)	1.433 (0.371–5.543)	1.536 (0.391–6.036)	1.229 (0.314–4.807)	1.486 (0.361–6.121)	1.270 (0.319–5.052)	1.624 (0.382–6.905)	1.237 (0.291–5.259)	1.293 (0.295–5.664)
Serum total PSA	1.072 (0.943–1.219)	0.240	57.7% (42.2–73.3)	1.075 (0.938–1.231)	1.057 (0.926–1.208)	1.082 (0.943–1.243)	0.932 (0.789–1.102)	1.068 (0.930–1.226)	0.917 (0.774–1.087)	0.949 (0.801–1.124)	0.935 (0.787–1.112)
Biopsy Gleason sum	1.375 (0.524–3.610)	0.519	53.9% (41.6–66.2)	0.932 (0.331–2.627)	1.010 (0.354–2.883)	0.931 (0.326–2.659)	0.907 (0.322–2.553)	0.986 (0.343–2.832)	1.017 (0.354–2.925)	0.903 (0.315–2.593)	0.970 (0.334–2.818)
PCA3 score	1.019 (1.002–1.036)	0.004	70.8 (58.9–82.6)	-	1.016 (0.999–1.034)	-	-	1.013 (0.996–1.030)	1.017 (0.999–1.035)	-	1.014 (0.996–1.032)
T2 score	1.004 (0.999–1.009)	0.025	62.0% (49.0–75.1)	-	-	1.004 (0.999–1.010)	-	1.003 (0.998–1.009)	-	1.004 (0.998–1.009)	1.003 (0.997–1.008)
PHI	1.050 (1.008–1.093)	0.004	71.7% (58.7–84.8)	-	-	-	1.068 (1.018–1.122)	-	1.068 (1.017–1.121)	1.066 (1.016–1.118)	1.065 (1.015–1.118)
AUC of multivariate models				68.9% (57.2–80.5)	74.4% (63.5–85.4)	71.5% (59.9–83.1)	76.0% (62.6–89.4)	75.2% (64.5–85.9)	81.1% (69.2–93.0)	79.2% (69.2–93.0)	82.7% (71.6–93.8)
Gain in predictive accuracy *				-	+5.5	+2.6	+7.1	+6.3	+12.2	+10.3	+13.8
*p* value *				-	0.056	0.428	0.240	0.052	0.028	0.076	0.011

All variables were treated as continuous variables except DRE (digital rectal examination; suspicious *vs.* non suspicious) and Gleason sum at biopsy (6 *vs.* ≥7). *: as compared with the base model. AUC: area under receiver operating curves; OR: odds ratio; PCA3: prostate cancer gene 3; PHI: Prostate Health Index; PSA: prostate-specific antigen; T2: TMPRSS2:ERG fusion gene.

In summary, we demonstrated that PHI and T2 and PCA3 scores correlated with tumor volume at prostatectomy. PHI correlated with Gleason score. T2 score proved to predict extracapsular extension while PCA3 score was able to predict multifocal prostate cancer. Association of PHI and PCA3 score was the most performing association.

### 2.4. Discussion

In the current study, we investigated the relationship between three biomarkers—PHI, urinary PCA3 and T2 scores—and PCa characteristics at final pathology in a population of patients treated with radical prostatectomy for localized PCa. The results of the study supported the hypothesis that each biomarker is able to predict some of the pathological disease characteristics, namely tumor volume ≥0.5 mL, extracapsular extension (≥pT3 tumors), multifocality, and Gleason sum. Of interest, while tumor volume is predicted by both PCA3 and T2 scores and PHI, the three tests predict specifically different characteristics, PCA3 score being indicative of multifocality, T2 score of extracapsular extension and PHI of Gleason sum.

Smaller tumors are thought to be less aggressive and less frequently associated with progression [37]. That is why Nakanishi *et al.* assessed the ability of PCA3 score to predict tumor volume at prostatectomy [23]. They observed a correlation, which was confirmed by several other published series whether the tumor volume was directly assessed on prostatectomy specimens [23,25,26,27,38] or indirectly by biopsy markers such as number or proportion of invaded cores, or length or proportion of invaded tissue (review in [5]). Two previous studies also suggested a correlation between PHI and tumor volume whether this correlation was evaluated on the basis of the number of positive cores at prostate biopsy [18] or the prostatectomy results [36]. Our results further support the relationships between both biomarkers and tumor volume. We failed however to find such a correlation between tumor volume and T2 score. Two studies previously evaluated this correlation in regards to prostatectomy results [12,32]. In the largest study (*n* = 187), Tomlins *et al.* found a correlation between T2 score and tumor volume while Young *et al.* did not (*n* = 41 specimens in which tumor volume was assessed by summed total linear dimensions) [32].

Extracapsular extension is also an obvious and major prognosis-related pathological feature [39]. Correlation with extracapsular extension has been reported for PHI [36], as well as for PCA3 [25,26,33] and T2 scores [30,35]. We could only confirm this relationship for T2 score, a result consistent with a recently published meta-analysis [9]. When evaluating such relationship in specimens from prostatectomies (8003 patients), biopsies (423 patients) or transurethral resections (350 patients), this meta-analysis indeed found that men with TMPRSS2:ERG fusion-positive cancers were more likely to have advanced stage tumors (T3 or greater *vs.* T2 or lower) [9]. Conversely to some studies [25,26,33], we did not observe any association between the PCA3 score and extracapsular extension. The lack of correlation was previously reported [23,24,28,29,30,31,34,38], although not explained.

As another pathological characteristic likely to influence treatment decision between radical or focal therapies [39], we tested PCa multifocality as an outcome to predict by PCA3 or T2 score or PHI. We previously reported the first study establishing a correlation between the PCA3 score and multifocality [27] and here confirm the results in a larger cohort. Another study found similar results with PCA3 test but failed, as in our results, to find relationship between tumor focality and T2 score [32].

In recent years, several efforts have been made to find biomarkers or combination of biomarkers that could help clinicians preoperatively determine PCa pathological characteristics and aggressiveness and therefore choose the best treatment option based on these parameters. In relation with the widespread use of PSA for individual screening, an increasing number of men are diagnosed with lower stage of PCa. These patients are consequently offered numerous options including radical prostatectomy and radiotherapy, but also prostate-sparing managements such as active surveillance and focal therapy. The use of a reliable, robust and accurate biomarker or a combination of such biomarkers could be dramatically helpful in clinical decision making. Such biomarkers could also be used as indicators of whether additional pretreatment examinations (prostatic MRI for example to assess extracapsular extension [40]) is warranted before making a definitive decision. These major clinical considerations prompted us to evaluate whether incorporating PHI, PCA3 score, and/or T2 score into the regression base model improved the accuracy of the prognostic model and therefore the clinical management. We showed that all three can improve predictive accuracy when compared to a base model that reflects clinical practice by including clinico-biological criteria and Gleason sum at biopsy. The decision strategy can, therefore, be meaningfully incremented by incorporating these biomarkers, in particular because they provide significant prediction for different aspects of PCa aggressiveness. This is of importance because the real interest in associating PHI, and PCA3 and T2 scores has not been established when evaluated for diagnostic purposes [18,19,30].

Our study presents some limitations, such as the relatively small size of our cohort, the inclusion of Caucasian only patients, or the fact that pathological examinations were not performed by a unique reference pathologist even all were experienced in the prostate pathology field. Results should therefore be considered with caution before applying them to all populations. Nevertheless, with well-documented and no missing data, data collection proved to be reliable and prediction models are likely to be robust.

## 3. Experimental Section

### 3.1. Patients and Study Design

From February 2008 to April 2012, 154 patients with informative preoperative urine sample (>10,000 copies/mL PSA RNA) and radical prostatectomy because of biopsy-proven PCa were prospectively included in the Department of Urology at Hospices Civils of Lyon. Prior to biopsy, the patients underwent clinical and biological evaluation including digital rectal examination (DRE) and transrectal ultrasonography (TRUS). Urine samples were obtained at that time and the mean interval between serum and urine sampling and prostatectomy was 4.2 months. Patients who received 5alpha-reductase inhibitors prior to urine sampling and surgery were excluded. None received neoadjuvant treatment. The institutional review board approved this study and all patients provided written informed consent to participate.

First voided urines were collected and transferred into a specific transport tube (Progensa^®^ PCA3 Urine Specimen Transport Kit, San Diego, CA, USA) after attentive DRE [4]. Samples were stored at −80 °C until use. PCA3 and PSA RNA were quantified in our lab (Progensa^®^ PCA3 Assay, Hologic Gen-Probe) [4]. Determination of T2 score was performed by Hologic Gen-Probe (San Diego, CA, USA), using the second-generation TMA assay as described [12]. The PSA mRNA levels determined in our lab to calculate PCA3 scores and the ones determined by Hologic Gen-Probe to calculate T2 scores were strongly correlated: *p* < 0.001 using linear regression, ρ coefficient = 0.974 and *p* < 0.0001 using Spearman test. PCA3 and T2 scores were calculated as the ratio of PCA3 or TMPRSS2:ERG to PSA RNA ×1000 or 100,000, respectively. Access^®^ 2 Hybritech p2PSAs were measured on the Access Immunoassay Systems (Beckman Coulter, Brea, CA, USA) as recommended [41], in combination with measurements of total and free PSA levels. The PHI was calculated using the formula PHI = ([-2]proPSA/free PSA) × √(total PSA). PHI, PCA3, and TMPRSS2:ERG analyses were performed without knowing pathological results.

### 3.2. Tissue Samples

The prostate was weighted after removing the seminal vesicles and measured in 3 dimensions. Prostate volume was calculated using the formula for elliptical volume: π/6 × height × width × length. All specimens were inked and processed using the standard technique. The apical segment, basal portion, bladder neck and seminal vesicles were handled separately. Each remaining prostate specimen was formalin fixed, paraffin embedded and sectioned at regular intervals in a transverse plane perpendicular to the posterior surface. Radical prostatectomies were examined by pathologists all experienced with prostate disease and blinded to PCA3 and T2 scores and PHI results. Pathological tumor staging used 2002 UICC TNM classification, 6th edition. The product of the 3 tumor dimensions was multiplied by 0.4 to estimate volume, as recommended by Chen *et al.* [42].Total tumor volume was determined by adding the volume of each tumor focus. Microscopic tumor foci were numbered but considered to have negligible volume. If more than 1 tumor was reported, the highest GS was used.

### 3.3. Data Analysis and Statistics

The primary end point of the study was to determine the accuracy of PCA3 score, T2 score and PHI to predict GS, tumor volume, multifocality, and extracapsular extension. Medians were compared using Mann-Whitney test. Spearman test and linear regression analyses were done to determine associations between PSA mRNA levels as quantified in our department for PCA3 score calculation and in Hologic Gen-Probe lab for T2 score calculation. Univariate logistic regression models addressed each outcome of interest. A base model was defined by the combination of age (continuous variable), DRE findings (suspicious *vs.* non-suspicious), serum PSA (continuous variable) and Gleason score (≤6 *vs.* ≥7). To evaluate whether incorporating PCA3 score and/or T2 score and/or PHI into the regression base model improved the accuracy of the prognostic model and therefore the clinical management, comparison of regression models or areas under curves and decision curve analyses (DCA) [43,44] were performed. Indeed, DCA examined the theoretical relationship between the threshold probability of the outcome of interest (GS, extracapsular extension, tumor volume ≥0.5 mL, multifocality) and the relative value of false-positive and false-negative results [43,45]. In our study, this analysis estimated the magnitude of benefit resulting from altering clinical management in patients with different threshold probabilities of PCa, as previously used [36]. Data were analyzed using software package STATA^®^ v11.0 (College Station, TX, USA) with *p* < 0.05 considered statistically significant.

## 4. Conclusions

We showed that PHI and urinary PCA3 and T2 scores are predictors of PCa pathological characteristics at radical prostatectomy such as tumor volume <0.5 mL, extracapsular extension and tumor multifocality. PHI and PCA3 and T2 scores could complement each other since they predict different pathological characteristics.
